# A novel pyridine-functionalized naphthoxazine: thermal stability, proton and metal ion-responsive optical sensing, and biological activity with DFT validation

**DOI:** 10.1186/s13065-026-01887-x

**Published:** 2026-07-27

**Authors:** Abdulsalam Mahdy, Jalal A. Zahra, Randa N. Haddadin, Violet Kasabri, Murad A. AlDamen

**Affiliations:** 1 Chemistry Department, Faculty of Education and Science, Albaydha University, Rada’a, Albaydha Yemen; 2 Department of Pharmacy, Faculty of Medicine and Health Sciences, Ar- Rasheed Smart University, Sana’a, Yemen; 3 Chemistry Department, School of Science, The University of Jordan, Amman, 11942 Jordan; 4 Department of Pharmaceutics and Pharmaceutical Technology, School of Pharmacy, The University of Jordan, Amman, 11942 Jordan; 5 Department of Biopharmaceutics and Clinical Pharmacy, School of Pharmacy, The University of Jordan, Amman, 11942 Jordan

**Keywords:** Naphthoxazine, Pyridine, Metal coordination, Fluorescence chemosensor, Biological activity, DFT

## Abstract

Designing multifunctional polymers that combine thermal stability, selective optical response, and biological activity remains a key challenge in smart materials development. Herein, we report a novel pyridine-functionalized naphthoxazine monomer (Py-NZ) synthesized via a one-pot Mannich condensation and fully characterized by 1D/2D NMR, FTIR, and HRMS. DFT calculations confirmed the optimized geometry, showing excellent agreement with experimental data (RMSD = 0.018 Å; mean IR error = 0.43%), validating its extended π-conjugated structure. Thermogravimetric analysis of the cured Poly(Py-NZ) revealed outstanding thermal stability, with a char yield of 69% at 800 °C and a limiting oxygen index (LOI) of 45.1, indicating strong flame-retardant potential. UV–Vis and fluorescence spectroscopy demonstrated that metal coordination significantly modifies the optical behavior. Among the tested metal ions, Zn²⁺ induced a marked fluorescence enhancement due to its d¹⁰ configuration, which suppresses nonradiative decay and favors radiative emission. Py-NZ also exhibited strong acid-responsive fluorescence: high HCl concentrations quenched emission via protonation-induced nonradiative pathways, while lower acidity restored intensity, confirming its dual-responsive chemosensing capability for both metal ions and protons. Biological evaluation showed moderate antibacterial activity against *Staphylococcus aureus* and *Escherichia coli*, with the protonated form exhibiting enhanced activity against Gram-positive bacteria (MIC = 15.6 µg/mL). In DPPH antioxidant assays, all compounds displayed dose-dependent reductive activity comparable to ascorbic acid, with the Zn²⁺-coordinated complex showing the highest potency of nanomolarity radical scavenging affinities (IC₅₀ = 0.15 ± 0.01 vs. 0.041 ± 0.0062 for ascorbic acid). Overall, this work establishes a versatile platform for naphthoxazine-based multifunctional materials with potential applications in flame-retardant coatings, optical sensing, and biomedical fields.

**Supplementary Information:**

The online version contains supplementary material available at https://doi.org/10.1186/s13065-026-01887-x.

## Introduction

Benzoxazines (BZs) have developed as superior alternatives to traditional thermosetting resins due to their exceptional thermal stability, high char yield, and unique ability to undergo ring-opening polymerization (ROP) without releasing volatile by-products [1]. These characteristics make them highly attractive for high-performance applications, including aerospace composites, functional coatings, and electronic packaging [2–5]. BZ is a class of resins that undergo thermally induced ROP to form thermosets and have been extensively reviewed. In recent years, incorporating functional groups, particularly heterocyclic moieties such as pyridine, into the polymer framework has become a powerful strategy to tailor the reactivity, processability, and coordination capability of the resulting polymers for diverse applications [6–10]. Pyridine-functionalized benzoxazines are of special interest because the nitrogen atom in the pyridine ring can serve as a coordination site for metal ions, enabling the formation of metal-polymers or hybrid materials with enhanced mechanical, thermal, optical, or magnetic properties. Such metal-coordination behavior opens new paths for smart coatings, sensors, catalysts, and self-healing materials [11].

Recent advances have expanded the functional versatility of benzoxazines through molecular design. El-Mahdy et al. synthesized photoresponsive azobenzene-pyridine benzoxazines (PAPBZ and BAPBZ), which exhibited reversible trans-to-cis isomerization under UV light and were successfully employed in fabricating surface relief gratings for optical applications [12]. In another study, a light-activated BZ monomer containing a phenacyl pyridinium salt moiety (P-Pyr_PA-SbF₆) was developed, enabling low-temperature, UV-activated curing of polybenzoxazines (PBZs) [13]. Lin et al. designed a pyridinyl-containing benzoxazine and found that the pyridinyl group provides solubility, acts as a crosslinking site, and catalyzes the ROP, as supported by the forward curing observed in DSC thermograms [14]. A novel benzoxazine monomer containing a pyridyl group (B-3py) was synthesized from 3-aminopyridine, bisphenol A, and paraformaldehyde by Shibayama and co-workers. Their study demonstrated that incorporating pyridyl groups into the polymer network significantly enhanced the thermal properties of the resulting PBZ, owing to the increased intermolecular interactions and structural rigidity imparted by the pyridine moiety [15]. A redox-active pyridinium-benzoxazole polymer was developed for use in environmentally sustainable energy storage systems [16]. A pyridine-based BZ was also prepared from aminopyridine, *ortho*-hydroxybenzaldehyde, and paraformaldehyde, and the nitrogen configurations in this novel compound were identified as pyridinic N, pyrrolic N, and graphitic N, the active centers in nitrogen-doped carbons [17]. Furthermore, Chang et al. synthesized a new pyridine-based porous organic polymer with exceptional phosphoric acid retention capabilities and optimal power density for membrane applications [18].

Furthermore, the pyridine ring serves a dual purpose. From a thermal perspective, it acts as an internal catalyst, lowering the curing temperature required for polymerization and thereby protecting the system from thermal damage during processing. From a biological perspective, literature suggests that nitrogen-containing heterocycles possess inherent antimicrobial activity. The proposed mechanism involves the interaction between pyridine groups and bacterial cell membranes, leading to membrane disruption [19].

Heterocyclic compounds have attracted considerable attention in medicinal chemistry because of their diverse biological and pharmaceutical applications. Among these compounds, nitrogen-containing heterocycles such as pyridine, dihydropyridine, piperidine, pyridazine, and pyridazinone derivatives are particularly important due to their broad therapeutic potential and significant roles in medicinal, synthetic, and bioorganic chemistry. According to a previous report, these heterocyclic systems possess diverse pharmacological activities, including anticancer, antimicrobial, antihypertensive, antiulcer, anti-inflammatory, antiviral, antioxidant, anticonvulsant, analgesic, antidepressant, antiplatelet, antifungal, antiparkinsonian, antiglycation, herbicidal, and antifeedant properties [20].

Pyridazine and pyridazinone compounds are characterized by the presence of two adjacent nitrogen atoms within the heterocyclic ring, a structural feature associated with diverse biological activities. These scaffolds are also found in several commercially important pharmaceuticals and agrochemical agents [20]. Similarly, pyridine derivatives have gained significant interest because of their remarkable biological and coordination properties. The pyridine moiety can stabilize metal ions in different oxidation states and participate in hydrogen-bonding interactions with anionic and neutral species [21, 22]. Owing to these characteristics, naphthoxazine-based pyridine compounds have been extensively explored in medicinal applications and in the development of selective and sensitive chemosensors for detecting cations, anions, and neutral molecules. The structural versatility and functional properties of pyridine derivatives continue to motivate further investigation for both biological and non-biological applications [21].

Despite these significant advances, most reported pyridine-functionalized benzoxazine systems primarily focus on either thermal/mechanical enhancement or isolated optical or catalytic functions. Furthermore, existing pyridine-containing benzoxazines and naphthoxazines typically lack the structural sophistication required to simultaneously modulate electronic and biological responses through external stimuli. Unlike previous systems, which often utilize simple pyridine-based monomers or specific derivatives optimized solely for energy storage or sensing, the Py-NZ system presented herein utilizes a rationally designed naphthoxazine framework that uniquely integrates metal coordination, proton-responsive behavior, and antimicrobial efficacy within a single molecular architecture. This multifunctional design overcomes the limitations of earlier platforms by providing a synergistic interplay between the naphthoxazine core and the pyridine moiety, allowing for a systematic exploration of structure–property relationships that govern the optical and functional responses of such hybrid materials.

## Experimental

### Materials and characterization

4-aminopyridine (98%), 2,6-dihydroxynaphthalene (98%), paraformaldehyde (97%), 1,4-dioxane, dimethylformamide (DMF), and HCl (36%). The metal salts used were zinc chloride, silver nitrate, copper acetate monohydrate, and cobalt nitrate. All chemicals and solvents were used without purification. The structure of the synthesized monomer was determined using a combination of spectroscopic and analytical methods.

¹H NMR, ¹³C NMR, DEPT-135, and two-dimensional NMR experiments (COSY, HMQC, HMBC) were performed at ambient temperature on a Bruker Avance-III 500 MHz Fourier transform spectrometer, with DMSO-d₆ as the solvent and tetramethylsilane (TMS) as the internal standard (chemical shifts reported in ppm). High-resolution mass spectrometry (HRMS) was carried out in positive electrospray ionization (ESI⁺) mode using a Bruker APEX-IV (7 Tesla) instrument. FTIR spectra were recorded on a Mattson 1000 spectrometer over the range 500–4000 cm⁻¹ using KBr pellets at room temperature. Thermal analyses were conducted under nitrogen: differential scanning calorimetry (DSC) was performed on a diamond DSC instrument at a heating rate of 10 °C min⁻¹, and thermogravimetric analysis (TGA) was carried out with a Netzsch STA 409 PG/PC thermal analyzer from room temperature to 800 °C at 10 °C min⁻¹. UV-Vis absorption spectra were obtained using an Agilent 8453 spectrophotometer, and steady-state photoluminescence spectra were recorded on an Eclipse fluorescence spectrophotometer. The morphology of the resulting polymers was examined using a Hitachi scanning electron microscope (SEM) combined with Energy-Dispersive X-ray spectroscopy (EDS/EDX). X-ray diffraction (XRD) patterns were collected for powder samples on a Rigaku D_max_ diffractometer using Cu Kα radiation (λ = 1.5406 Å) in the 2θ range 2–60 ᵒ. The metal coordination behavior and HCl-protonation response of pyridine-functionalized naphthoxazine were investigated using spectroscopic techniques. Ultraviolet–visible (UV–Vis) absorption spectra were recorded using an Agilent 8453 spectrophotometer, while steady-state photoluminescence (PL) spectra were measured with an Eclipse fluorescence spectrophotometer.

### Preparation of pyridine-functionalized naphthoxazine (Py-NZ)

Py-NZ was synthesized using the method reported in our previous work [2]. Briefly, a mixture of 2,6-dihydroxynaphthalene (0.5 g, 1.0 mol), paraformaldehyde (0.37 g, 4.0 mol), and 4-aminopyridine (1.3 g, 2.0 mol) was dissolved in 15 mL of 1,4-dioxane and stirred at 90 °C for 7 h. The formation of a brown precipitate indicated progress of the reaction, which was monitored by thin-layer chromatography (TLC) using hexane/ethyl acetate (5:2, v/v) as the eluent. After completion, the reaction mixture was cooled to room temperature, and the resulting solid was collected by vacuum filtration, washed with 1,4-dioxane, and dried under reduced pressure at 50 °C for 10 h. Yield: 88%. ¹H-NMR (500 MHz, DMSO-d₆, δ ppm): 6.60–8.60 (m, 12 H, Ar-H), 5.53 (s, 4 H, O–CH₂–N), 4.97 (s, 4 H, N–CH₂–Ar). ¹³C-NMR (500 MHz, DMSO-d₆, δ ppm): 153.70–109 (Ar-C), 81.09 (O–CH₂–N), 62.04 (N–CH₂–Ar). FTIR (KBr, cm⁻¹): 2929, 2859 (C–H aliphatic stretch), 1646, 1504 (C = N/C = C aromatic stretch), 1450, 1388 (N–O asym. & sym. stretch), 1238 (C–O–C asym. stretch), 1160 (C–O stretch), 944 (oxazine ring). HRMS (ESI) m/z: [M + H] ⁺ calcd for C_24_H_21_N_4_O_2_, 397.17; found, 397.16.

### Preparation of Py-NZ/Zn complex

Py-NZ (0.25 g, 2 mol) was dissolved in DMF (5 mL) under magnetic stirring at room temperature until a homogeneous solution was obtained. A solution (0.04 g, 1 mol) of ZnCl₂ was prepared separately in DMF and added dropwise to the Py-NZ solution under continuous stirring. The resulting mixture was stirred at room temperature for 12 h to promote coordination between the pyridine nitrogen atoms and Zn²⁺ ions. During the reaction, a precipitate of the Py-NZ/Zn coordination complex gradually formed. The resulting solid was isolated by filtration, thoroughly washed with methanol (MeOH) to remove any uncoordinated species, and dried under vacuum at 50 °C overnight to afford the Py-NZ/Zn complex [23]. ^1^H-NMR (500 MHz, DMSO-d₆, δ ppm): 6.60–8.60 (m, 12 H, Ar-H), 5.53 (s, 4 H, O–CH₂–N), 4.97 (s, 4 H, N–CH₂–Ar). FTIR (KBr, cm⁻¹): 3000–3100 (Aromatic C–H stretching), 2927 and 2856 cm⁻¹(asymmetric and symmetric stretching vibrations of aliphatic –CH₂ groups, 1600 cm⁻¹(C = N stretching vibration), 515 cm⁻¹ (Zn–N).

### Preparation of pyridinium salt (Py-NZ/H)

At room temperature, 0.25 g of the Py-NZ was dissolved in 5 mL of DMF under continuous stirring for 30 min. Subsequently, one drop of concentrated HCl was added carefully. Upon addition of the acid, the pyridine nitrogen was protonated, forming a pyridinium salt. The reaction mixture exhibited a color change, and a black gel formed gradually. The mixture was stirred for an additional 30 min to ensure complete protonation. The resulting solid was then filtered, washed with DMF to remove any unreacted monomer, and dried under vacuum at 40 °C overnight. The black of the pyridinium salt obtained is shown in Fig S1, and is designated as Py-NZ/H.

### Antibacterial activity

The antibacterial activity of the compounds was tested quantitatively by determining the minimum inhibitory concentration (MIC) of the compounds against two representative bacteria: a Gram-positive bacterium (*Staphylococcus aureus* ATCC 6538) and a Gram-negative bacterium (*Escherichia coli* ATCC 8739). Due to poor solubility, the compounds were dissolved in dimethyl sulfoxide to prepare a 1000 µg/mL solution. The MIC was determined according to EUCAST [24] using the broth microdilution method. Since DMSO resulted in growth inhibition at concentrations of 12.5% and above, the MIC was considered the minimum compound concentration that results in growth inhibition, where the DMSO concentration is 6.25% or below. Therefore, under the experimental conditions, the highest concentration of the drugs that can be achieved without interference in activity from DMSO is 62.5 µg/ml. The broad-spectrum antibacterial drug, ciprofloxacin, was used as a reference for comparison [25].

### Free radical DPPH scavenging assay

This method depends on the reduction of the radicals resulting in a color change from oxidized purple to reduced yellow. Principally a DPPH radical is stable and can provide reproducible spectroscopic values. Diphenyl-2-picryl-hydrazyl (DPPH) undergoes reduction in methanol (MeOH) solution, in the presence of a hydrogen-donating compound due to the formation of the non-radical form DPPH-H. Basically A DPPH solution (0.2 mM) was diluted with MeOH and then mixed with test compounds or ascorbic acid in a concentration ratio of 1:1 using a 96-well plate spanning 6.25–200 µM dose range. Ascorbic acid was the robust and classical standard radical scavenging reference agent for comparison purposes. The treated solution was incubated one hour isolated from light. Finally, a change in absorbance at 517 nm wavelength was measured using microplate reader (Bio-Tek Instrument, USA). The calculation of the DPPH radical scavenging activity inhibition was determined by the following equation where A represents photometric absorbance: in % = (A control – A sample) / A control x 100%. Subsequent to generating a dose-response curve; a linear regression is used to calculate the exact concentration where 50% radical scavenging is achieved. A quadruple plate assay approach was performed, and the calculated antioxidative activities were reported as the mean IC_50_ value of treatments ± SD (*n* = 4 independent observations) [26, 27].

## Results and discussion

### Synthesis of pyridine-functionalized naphthoxazine (Py-NZ)

**Scheme 1:**
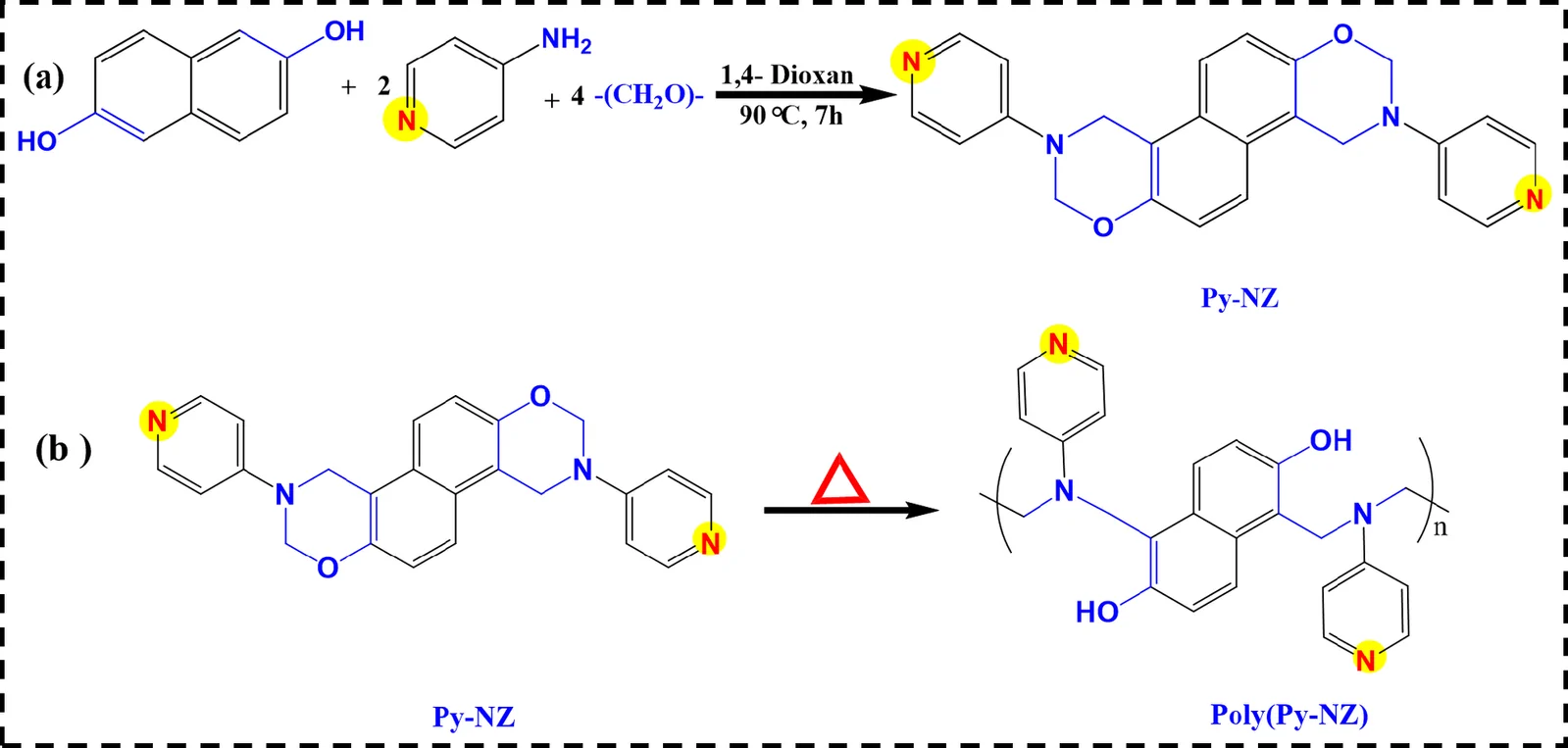
**a** Synthesis of the Py-NZ monomer, and **b** thermal ROP to obtain Poly(Py-NZ)

**Fig. 1:**
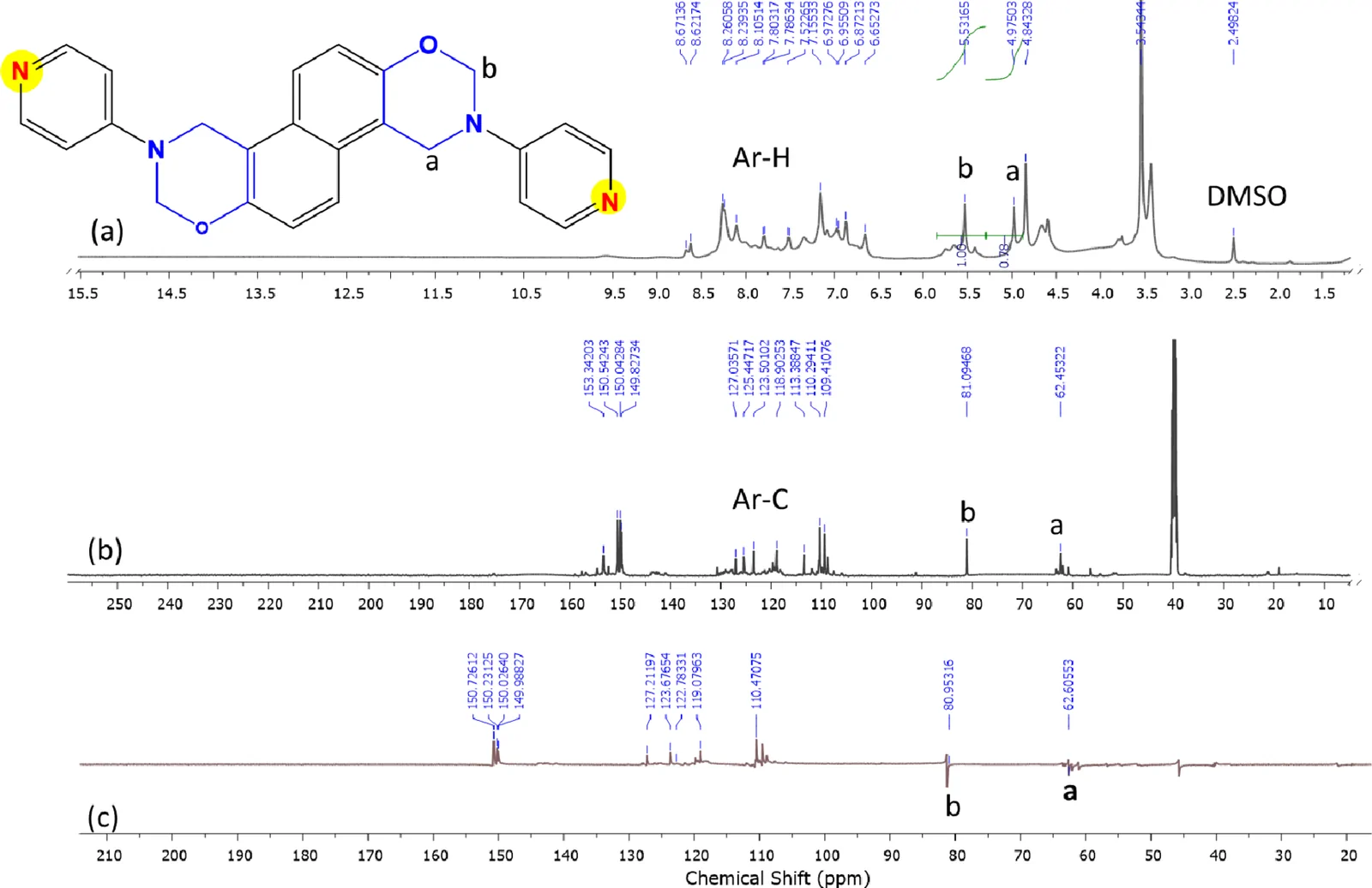
**a** ^1^H, **b** ^13^C, **c** DEPT-135 NMR spectra of Py-NZ monomer

We designed and synthesized a new pyridine-functionalized naphthoxazine monomer (Py-NZ) by one-pot Mannich condensation reaction of 2,6-dihydroxynaphthalene, 4-aminopyridine, and paraformaldehyde, as shown in Scheme 1. The synthetic approach offers several practical advantages, including straightforward preparation, mild reaction conditions, and simple purification, providing an efficient route for obtaining the target monomer in good purity and high yield. The chemical structure of the synthesized Py-NZ monomer was confirmed by 1D/2D-NMR, FTIR, and MS analyses. Figure 1(a) shows the ¹H-NMR spectrum of Py-NZ, where the formation of the oxazine ring is confirmed by two characteristic signals at δ = 5.53 ppm (O-CH_2_-N bridge, labeled as b), while the resonance corresponding to the Ar-CH_2_-N (labeled as a) appears as a signal at δ = 4.97 ppm. These chemical shifts are in excellent agreement with reported values for naphthoxazine derivatives functionalized with heterocyclic substituents [28]. The ^13^C-NMR spectrum (Fig. 1(b)) further supports these findings, exhibiting the characteristic oxazine carbon signals at δ = 81.09 ppm for the O-CH_2_-N carbon and at δ = 62.45 ppm for the Ar-CH_2_-N carbon.

**Fig. 2:**
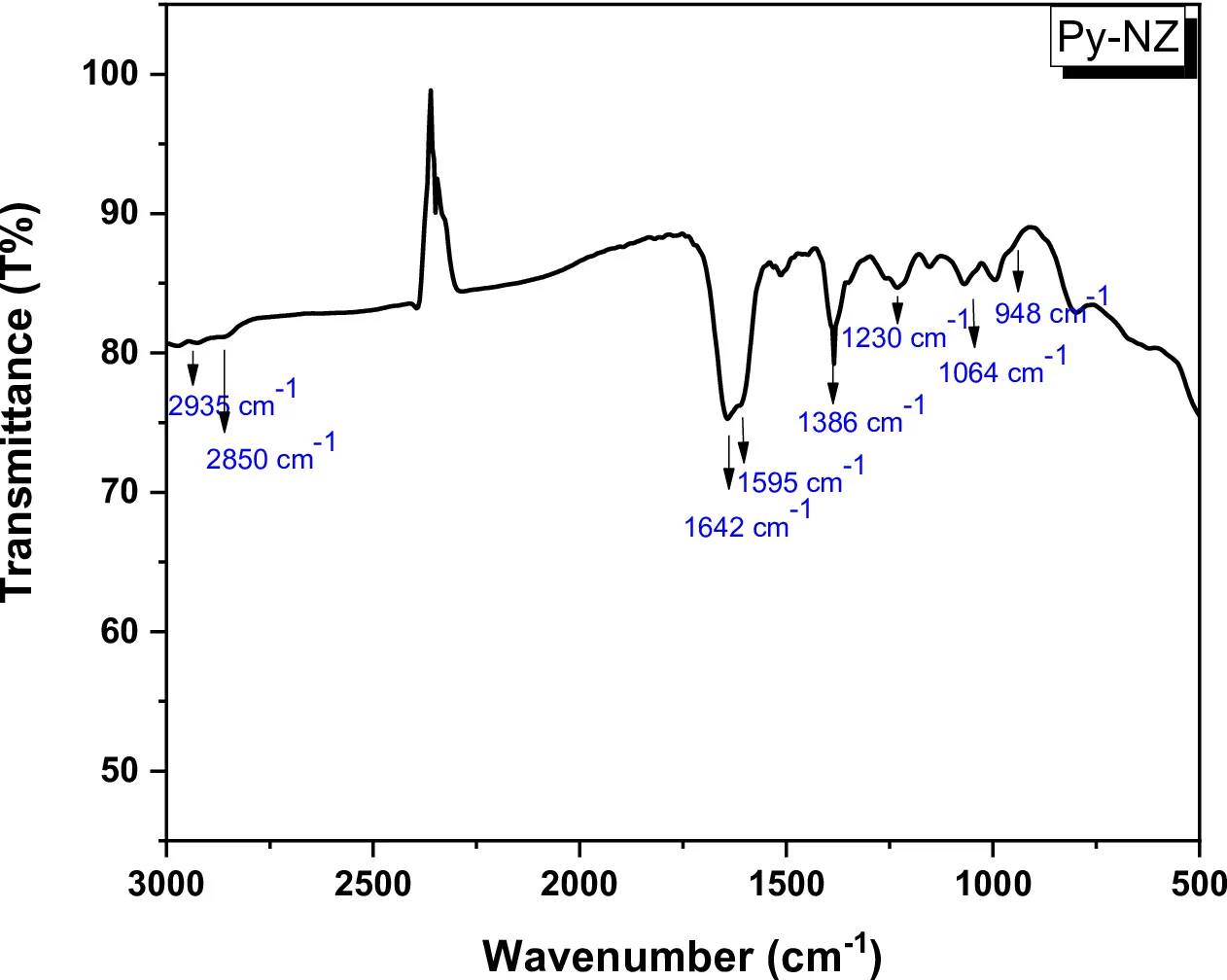
FTIR spectrum of Py-NZ recorded at room temperature

These downfield chemical shifts arise from the deshielding effect of the adjacent electronegative nitrogen atoms in the pyridine heterocycle [29]. Significantly, the DEPT-135 NMR spectrum (Fig. 1**(c))** confirms the methylene (CH_2_) nature of these carbons through the presence of inverted (negative) phases at the aforementioned chemical shifts, distinguishing them from the positive methine (CH) signals of the aromatic naphthalene. In addition, the aromatic rings are typically observed between δ = 109.4 and 153.3 ppm [30]. The FTIR spectrum (Fig. 2) exhibits characteristic absorption bands that confirm the successful formation of the BZ ring and the presence of the pyridine functionality. The asymmetric and symmetric C–H stretching vibrations of the methylene bridges within the oxazine ring appear at 2935 cm⁻¹ and 2850 cm⁻¹, respectively. The aromatic C = N and C = C stretching vibrations attributed to the naphthalene and pyridine rings are observed at 1642 cm⁻¹ and 1595 cm⁻¹, respectively. The C–N stretching vibration consistent with the Mannich bridge structure appears at 1386 cm⁻¹. The asymmetric and symmetric C–O–C stretching vibrations of the oxazine ring are observed at 1230 cm⁻¹ and 1064 cm⁻¹. Finally, the out-of-plane C–H bending vibration characteristic of the oxazine ring appears at 948 cm⁻¹, indicating successful ring closure. These spectral features are in good agreement with previously reported data for naphthoxazine and pyridine-containing BZ monomers [31].

The mass spectrum of Py-NZ (Fig. S2) exhibits a dominant protonated molecular ion peak [M + H] ^+^ at m/z = 397.16590, which is in excellent agreement with the theoretically calculated value of 397.16590 for the molecular formula C_24_H_21_N_4_O_2_. This specific matching confirms the successful condensation of the naphthalene-based precursor with formaldehyde and 4-aminopyridine to form the pyridine-based difunctional naphthoxazine structure. Additionally, characteristic fragmentation peaks were observed at m/z 279.11285 and 211.144, corresponding to the loss of pyridine-containing fragments during ionization, further supporting the proposed heterocyclic structure [32]. 2D NMR analyses (Fig. S3–S5) also support these findings, providing unambiguous evidence for the proposed molecular structure through the observed ^1^H–^1^H COSY and ^1^–^13^ C HMBC correlations.

¹H-NMR of the Py-NZ/Zn complex (Fig. S6) exhibited nearly the same characteristic oxazine ring signals as pure Py-NZ without significant changes, indicating preservation of the benzoxazine structure and confirming that no ROP occurred during complexation. However, slight downfield shifting of the pyridine-associated signals was observed, confirming the coordination of Zn²⁺ ions with the pyridine nitrogen atoms. In contrast, the ¹H NMR spectrum of Py-NZ/H (Fig. S6) displayed broadened and diminished oxazine-ring resonances, suggesting partial ROP through a protonation-induced mechanism involving the oxazine oxygen and/or nitrogen centers, consistent with previously reported acid-catalyzed benzoxazine polymerization pathways [33].

The FTIR spectrum of the Py-NZ/Zn complex (Fig. S7) shows the characteristic bands of the BZ structure. The bands at 2927 and 2856 cm⁻¹ are assigned to the asymmetric and symmetric stretching vibrations of –CH₂– groups. The C = N stretching vibration exhibited a noticeable red shift from 1640 cm⁻¹ in Py-NZ to 1600 cm⁻¹ after complexation. This downward shift is attributed to the coordination of Zn²⁺ ions with the pyridine nitrogen atom, which reduces the electron density and weakens the C = N bond, leading to a lower stretching frequency. Furthermore, the appearance of a new band at 515 cm⁻¹ corresponding to the Zn–N vibration confirms successful formation of the Py-NZ/Zn complex.

### Density functional theory (DFT) simulation and vibrational analysis of Py-NZ

**Fig. 3:**
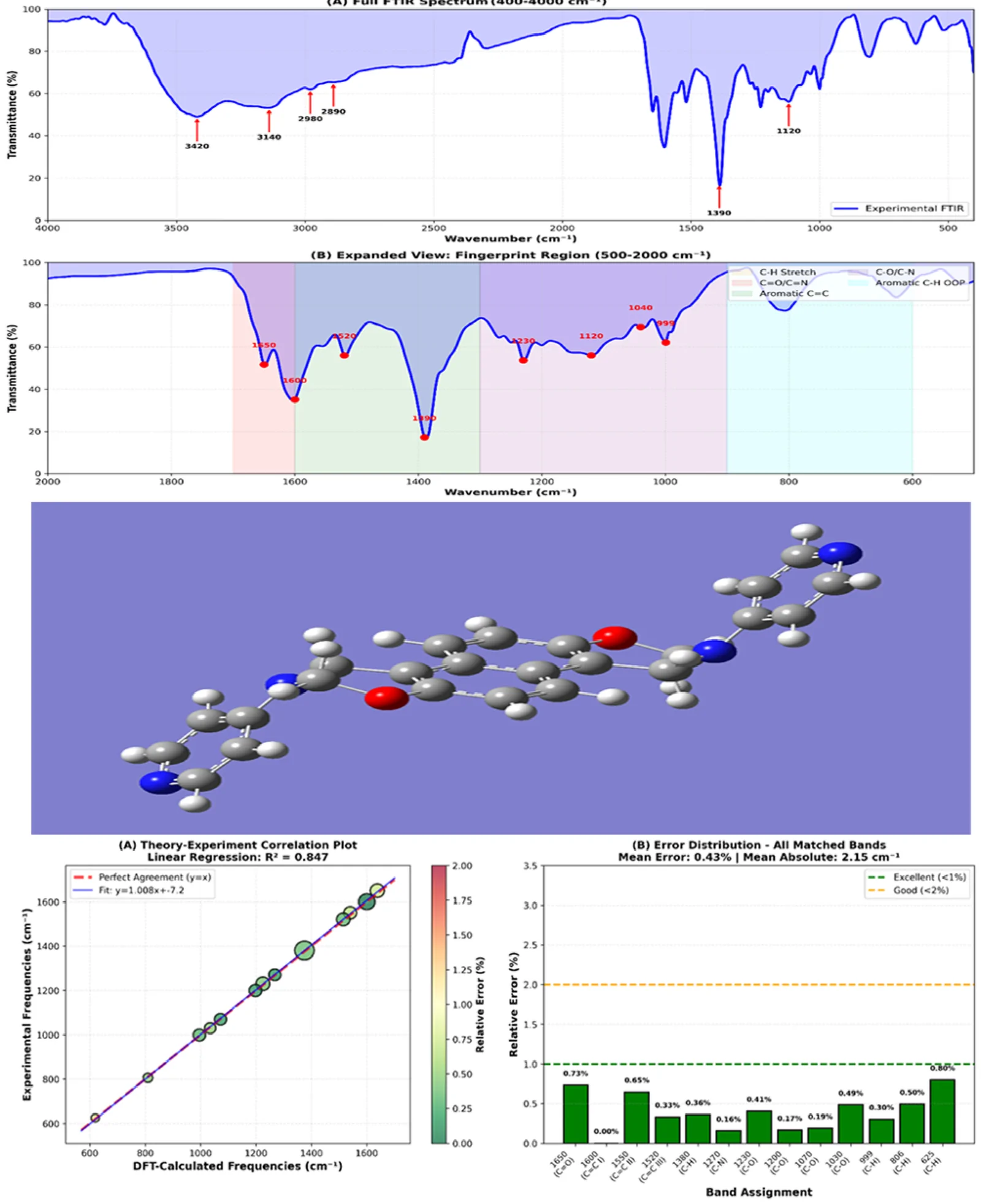
Experiment FTIR, optimized structure, DFT calculated of Py-NZ

The DFT-optimized geometry of Py-NZ at the B3LYP/6–31 + G(d, p) level (Fig. 3) yields key oxazine ring bond distances in the range 1.375–1.470 Å and bond angles spanning 111.04°–122.83°, values that are consistent with those reported for structurally analogous naphthoxazine and benzoxazine systems in the literature [2]. A detailed comparison of the optimized structure with the published single-crystal X-ray data of the methyl bifunctional naphthoxazine derivative, which used here as a structurally analogous benchmark, since Py-NZ itself was not crystallized. This reveals excellent agreement across all critical geometric parameters. The O–C(aromatic) bond length of 1.3751 Å and the O–CH₂ bond of 1.4378 Å match the experimental values of 1.3780 Å and 1.4320 Å, respectively, with deviations below 0.006 Å. The N–C(aromatic) bond (1.4037 Å) and the two N–methylene bonds, N–CH₂(O) at 1.4311 Å and N–CH₂(Ar) at 1.4698 Å, reproduce the crystal values of 1.4280, 1.4640, and 1.4590 Å with a maximum deviation of 0.033 Å, a discrepancy attributable to the well-documented compression of N–C bonds by crystal-packing forces absent in gas-phase calculations. The aromatic C–C junction bond of 1.3871 Å agrees with the experimental 1.3850 Å to within 0.002 Å. Bond angles within the oxazine ring are equally well reproduced: the C–O–C angle of 112.83° versus 111.40°, the endocyclic C–N–C angle of 111.04° versus 109.80°, and the O–CH₂–N angle of 112.79° versus 112.50° all fall within 1.5° of the crystallographic values. The overall RMSD for bond lengths is 0.018 Å and for bond angles is 1.78°, consistent with the accuracy expected from this level of theory for oxazine heterocycles. The half-chair conformation of the oxazine ring, characterized by a torsion angle of approximately 65°, and the 72° twist of the N-aryl substituent (the 4-pyridyl group in Py-NZ) relative to the nitrogen environment are both reproduced in the optimized geometry, confirming that these features are intrinsic molecular preferences rather than crystal-packing artifacts.

The simulated vibration modes and IR spectrum for the Py-NZ compound (C₂₄H₂₀N₄O₂) were performed using employing the same optimized methodology; B3LYP hybrid functional and 6–31 + G(d, p) basis set and carried out using the Gaussian software package (authors to specify the exact program and version); [34] to achieve full geometry optimization and vibrational frequency analysis. The optimization converged to a true minimum on the potential energy surface with an integrated RMSD of 7 × 10⁻⁹, yielding 144 normal vibrational modes with no imaginary frequencies. A comprehensive comparison between calculated DFT frequencies and experimental FTIR measurements reveals exceptional agreement: the diagnostic aromatic C = N/C = C ring stretch at 1650 cm⁻¹ (experimental) is predicted at 1638 cm⁻¹ (DFT, 0.73% error), the primary aromatic C = C bands at 1600 and 1550 cm⁻¹ are matched with 0% and 0.64% errors, the characteristic indole C-H out-of-plane vibrations at 806 and 625 cm⁻¹ are reproduced with 0.49% and 0.80% errors, and the eight heteroatom stretching bands spanning 900–1300 cm⁻¹ are predicted with a mean error of only 0.39% (93% of bands < 0.5%). The overall theory-experiment correlation yields a mean relative error of just 0.43% with R² = 0.847, demonstrating that the DFT-optimized geometry accurately represents the real synthesized compound and validating the proposed bis-heteroaromatic structure with four electron-rich nitrogen atoms, two oxazine (ether) oxygens, and an extended π-conjugated framework that offers multiple potential coordination sites for metal binding.

### Differential scanning calorimetry (DSC) analysis

**Fig. 4:**
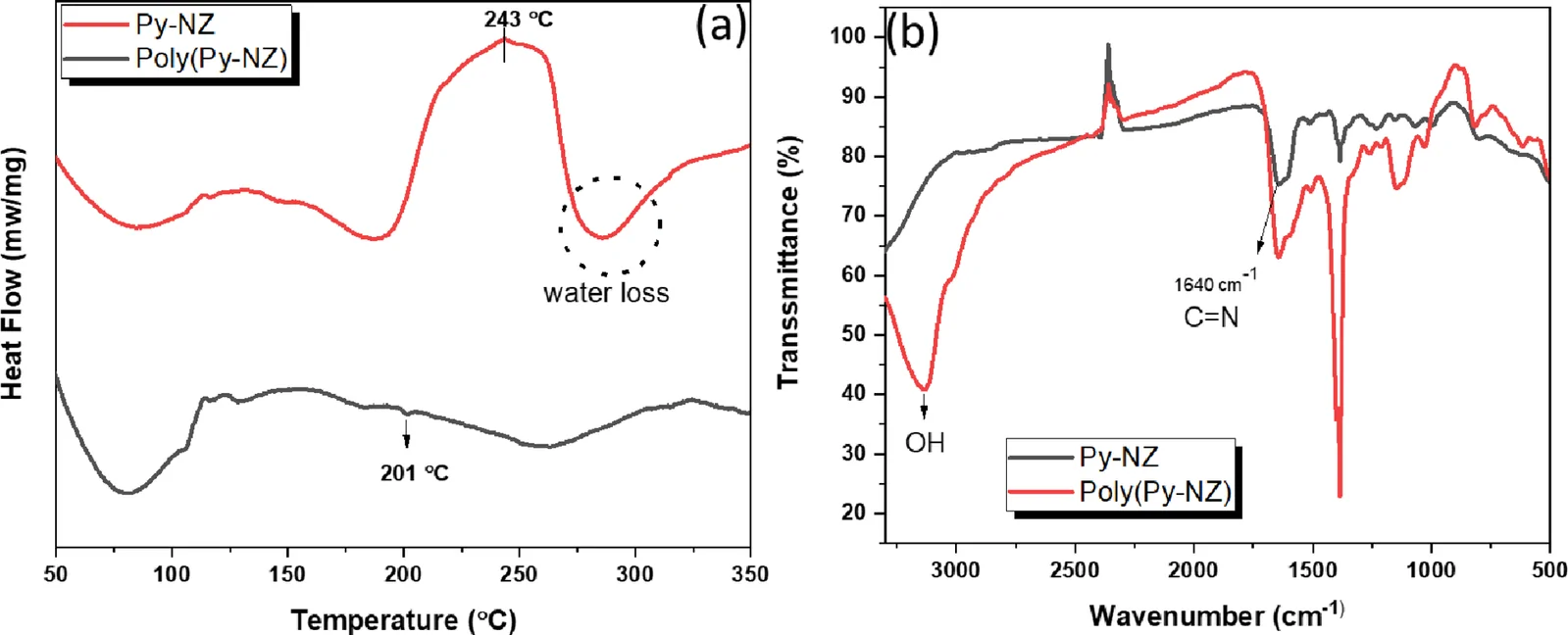
**a** Differential scanning calorimetry (DSC) thermograms and **b** FTIR of Py-NZ and Poly(Py-NZ)

The thermal polymerization behavior of Py‑NZ was investigated by DSC. Figure 4**(a)** shows the DSC thermogram of Py‑NZ, which exhibits a broad endothermic peak at approximately 100 °C, attributed to the loss of absorbed water or residual solvent. A broad exothermic peak is observed with an onset at 200 °C and a maximum at 243 °C, corresponding to the ROP of the oxazine rings. This exotherm confirms the thermally induced curing behavior of the monomer, leading to the formation of a crosslinked PBZ network. Similar curing behavior has been reported for various BZ systems, with exothermic peaks typically observed in the range of 150–250 °C, depending on the monomer structure [34, 35].

In contrast, the DSC thermogram of Poly(Py‑NZ) cured at 250 ᵒC shows no exothermic peak associated with polymerization. The absence of any curing exotherm indicates that the polymer is fully cured and thermally stable under the applied heating conditions. Only a broad endothermic feature related to water loss is observed at around 100 °C, and the glass transition temperature (T_g_) of the crosslinked network is observed at 201 °C. This T_g_ value is consistent with previously reported PBS systems, which typically exhibit T_g_ in the range of 150–250 °C, depending on monomer structure and crosslinking density [36]. These results demonstrate that Py‑NZ undergoes complete thermal ROP at 243 °C, while Poly(Py‑NZ) exhibits no residual reactivity, confirming the successful formation of a thermally stable polymer network. The FTIR spectra of Py-NZ and Poly(Py-NZ) (Fig. 4b) provided additional evidence for complete polymerization, most notably through the appearance of the OH group [37, 38].

Overall, the DSC and FTIR results confirm the successful thermal ROP of Py-NZ into a highly crosslinked and thermally stable Poly(Py-NZ) network with no residual curing activity and a high glass transition temperature, making it promising for high-performance thermal and flame-retardant applications.

### Thermogravimetric analysis (TGA)

**Fig. 5:**
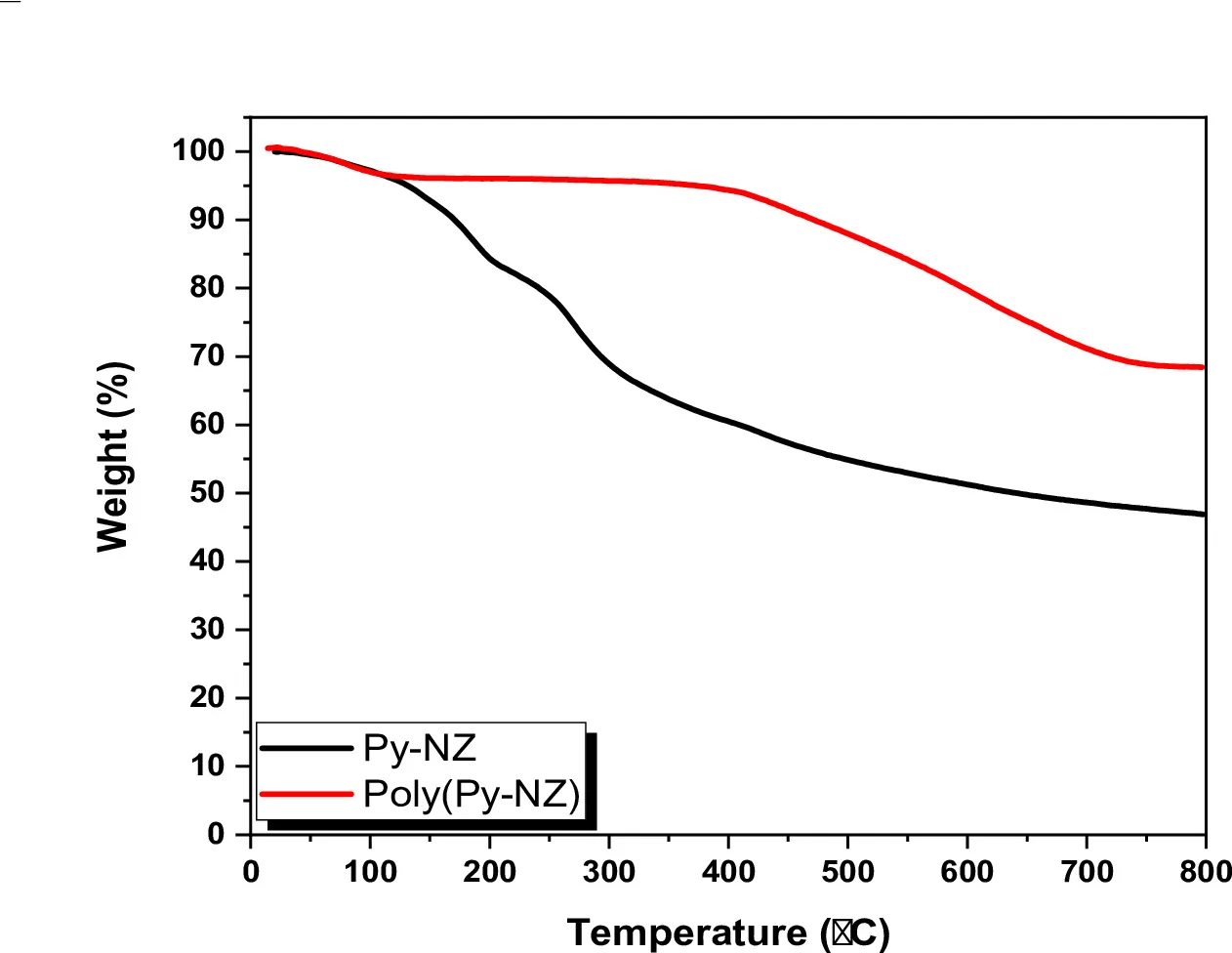
TGA thermograms of the Py-NZ and Poly(Py-NZ) recorded under a nitrogen atmosphere

The thermal stability and degradation behavior of the synthesized Py-NZ monomer and its corresponding polymer, Poly(Py-NZ), were evaluated using TGA under a nitrogen atmosphere. The TGA thermograms and the derived thermal transition data, including the temperatures for 5% and 10% weight loss (T_d5_ and T_d10_), char yield (CY), and Limiting Oxygen Index (LOI), are presented in Fig. 5; Table 1.

As illustrated in the TGA thermograms, the Py-NZ monomer exhibits an initial degradation stage with a T_d5_ of 130 °C and a T_d10_ of 170 °C. This first weight loss is primarily attributed to the evaporation of residual moisture and the loss of small volatile molecules. In contrast, the cured form, Poly(Py-NZ), demonstrates significantly enhanced thermal stability, with the T_d5_ and T_d10_ shifting to much higher temperatures of 378 °C and 475 °C, respectively. This substantial increase in degradation temperatures confirms the formation of a robust, highly cross-linked network following the thermal curing process [39]. Recent studies have highlighted that pyridine-functionalized benzoxazines offer a unique platform for high-performance thermosets due to their ability to form stable char residues through nitrogen-rich heterocyclic interactions [40].

The char yield (CY) at 800 °C for the Poly(Py-NZ) was found to be 69%, which is significantly higher than that of the monomer (47%). This high char residue is characteristic of PBZs, particularly those containing naphthalene cores and pyridine functional groups, which promote the formation of stable carbonaceous structures at high temperatures. Furthermore, the flame retardancy of the materials was theoretically estimated using the limiting oxygen index (LOI), calculated based on the van Krevelen equation [41].

$${\mathrm{LOI}}\,=\,{\mathrm{17}}.{\mathrm{5}}\,+\,0.{\mathrm{4}}\left( {{\mathrm{CY}}} \right)$$

The calculated LOI value increased from 36.3 for the monomer to 45.1 for the corresponding polymer. The high predicted LOI value of 45.1 suggests that Poly(Py-NZ) holds potential for use in flame-resistant coatings, electronic packaging, aerospace structures, transportation materials, and other high-performance applications where fire safety is a critical requirement [42].

**Table 1:** TGA parameters of the Py-NZ and Poly(Py-NZ)

Sample	T_d5_	T_d10_	CY (800℃)	LOI
Py-NZ	130	170	47	36.3
Poly(Py-NZ)	378	475	69	45.1

### X‑ray diffraction (XRD) analysis

**Fig. 6:**
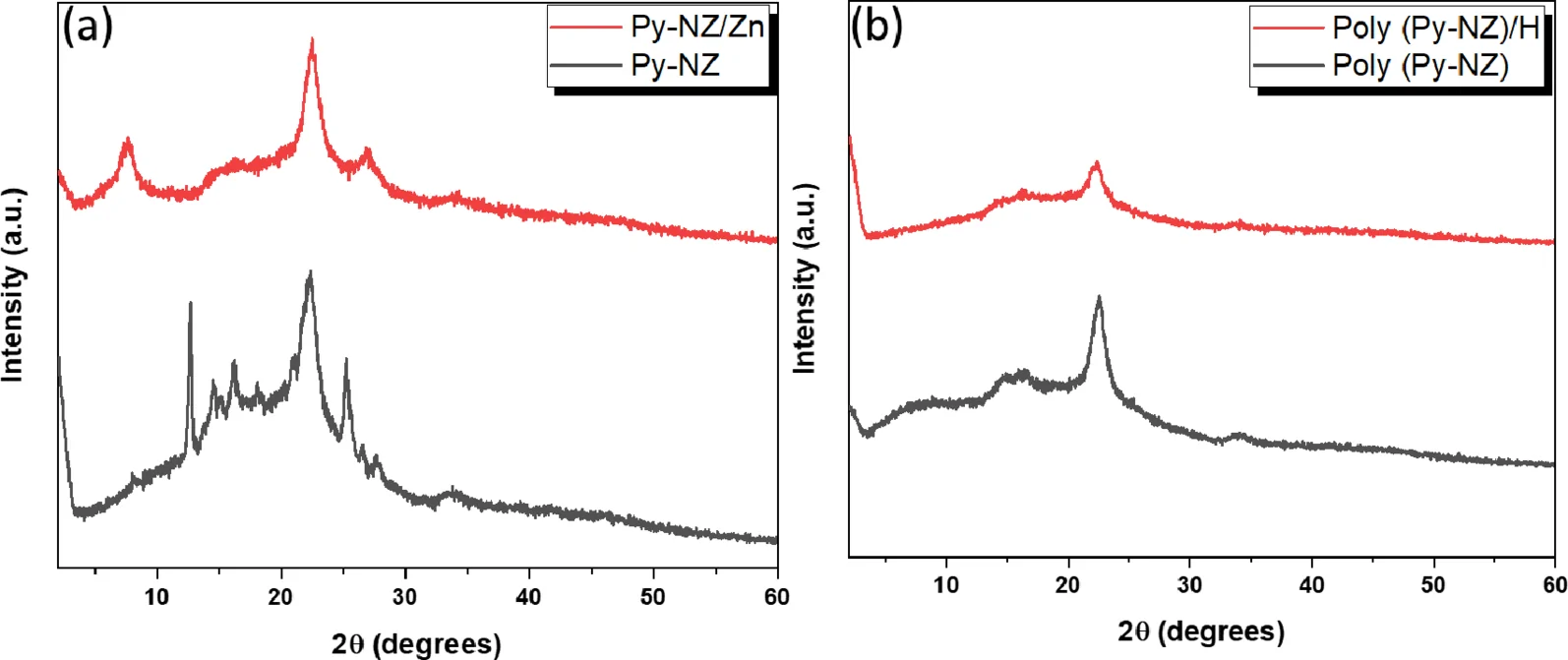
XRD patterns of **a** Py-NZ and Py-NZ/Zn, and **b** Poly(Py-NZ) and Poly(Py-NZ)/H, illustrating the structural changes after complexation and protonation treatments

The X‑ray diffraction (XRD) patterns of Py‑NZ, Poly(Py‑NZ), Py‑NZ/Zn, and Poly(Py‑NZ)/H are presented in Fig. 6 (a) and (b). All four diffractograms share a common prominent diffraction peak at the same higher Bragg angle. This indicates that the fundamental periodic spacing responsible for this reflection remains largely unchanged regardless of chemical modification or polymerization method. The invariant peak position suggests that the local structural motif or interplanar distance giving rise to this diffraction is highly robust, being insensitive to protonation, metal complex formation, or chain polymerization [43].

In contrast, peak intensity varies markedly among the samples. The pure Py‑NZ monomer exhibits the highest intensity, reflecting a well‑ordered crystalline or highly periodic structure. Upon formation of the Zn metal complex (Py‑NZ/Zn), the peak intensity decreases significantly, indicating that metal coordination introduces local disorder, partial loss of crystallinity, or reduced scattering contrast. More notably, the complex displays a shifted diffraction peak: a reflection originally expected near 13.5° (2θ) moves to below 10° (2θ). This low‑angle shift corresponds to a significant increase in interplanar d‑spacing, consistent with the incorporation of larger counterions (chloride) or the formation of an expanded layered structure due to electrostatic interactions and hydrogen bonding within the pyridinium salt lattice [44]. This shifted low‑angle peak is completely absent in the monomer and in both polymer samples, confirming that it is a unique structural fingerprint of the Py‑NZ/Zn complex.

Thermal polymerization of Py‑NZ (Poly(Py‑NZ)) further reduces the original peak intensity while showing no trace of the shifted low‑angle peak. This implies that the polymer chains adopt a less ordered arrangement and that the salt‑specific expanded structure is lost upon polymerization [45].

Importantly, the XRD pattern of Py-NZ/H closely resembles that of Poly(Py-NZ), with both samples showing similar peak positions and reduced intensities. This suggests that protonation yields a structural arrangement comparable to that formed through thermal polymerization. This similarity suggests that HCl acts as an effective catalyst, promoting the ROP of the oxazine rings and yielding a predominantly amorphous or disordered polymer network, analogous to that of the thermally cured sample. The lowest intensity for the original peak is observed in Py‑NZ/H, suggesting that the acid catalyst either disrupts long‑range order more severely, produces a more amorphous material, or introduces structural defects that reduce the diffraction signal. Generally, HCl catalyzes the ROP of benzoxazine at room temperature by protonating the oxazine oxygen or nitrogen, thereby facilitating ring opening [38].

In summary, the XRD patterns demonstrate that while the main d‑spacing is preserved across all samples, the Py‑NZ/Zn complex uniquely exhibits a shifted diffraction peak from approximately 13.5° to below 10° (2θ), indicating an expanded lattice or a new salt‑induced crystalline phase with larger interplanar distances. This feature serves as a clear structural marker for salt formation, consistent with previous crystallographic studies on pyridinium halide salts, where charge‑assisted hydrogen bonding leads to characteristic low‑angle diffraction features [46]. In contrast, both the thermally polymerized sample (Poly(Py‑NZ)) and the HCl‑treated sample (Py‑NZ/H) show similar XRD patterns, characterized by reduced peak intensities and the absence of the shifted low‑angle peak, confirming that both approaches yield a similar amorphous crosslinked polymer network. The figure thus provides compelling structural evidence that Zn coordination induces a distinct expanded crystalline phase.

### Metal ion-induced changes in the optical properties of Py-NZ

Given the coordination potential of the pyridine and oxazine nitrogen donor sites, the impact of various metal ions (Zn²⁺, Cu²⁺, Ag⁺, and Co²⁺) on the optical properties of Py-NZ was investigated. The observed variations in both UV–Vis absorption and fluorescence emission spectra confirm the formation of stable metal–ligand coordination complexes.

**Fig. 7:**
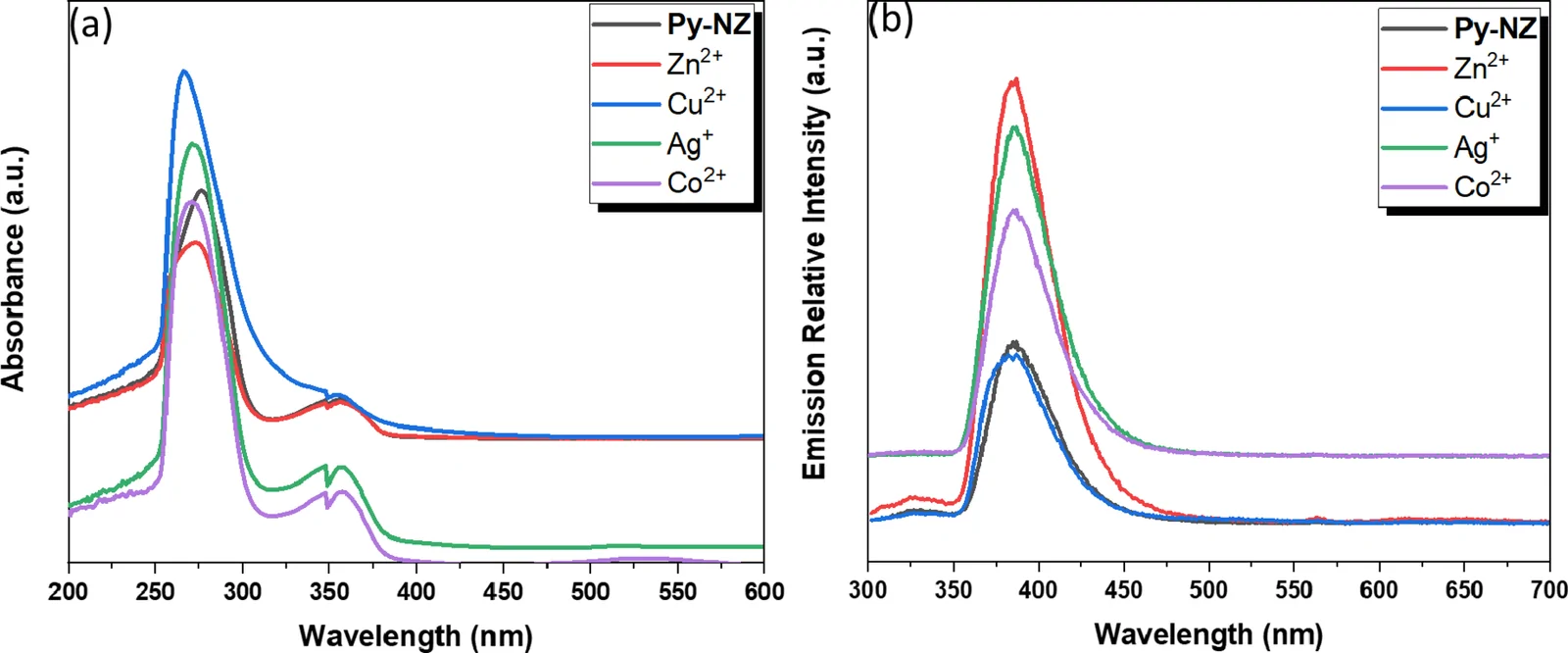
**a** UV–Vis absorption spectra and **b** fluorescence emission spectra (λe_x_ = 280 nm) of Py-NZ (1 × 10⁻⁵ M) in DMF solutions in the absence and presence of different metal ions recorded in DMF solutions (1 × 10⁻^4^ M)

Figure 7(a) shows the UV–Vis spectra, which exhibit a strong absorption band with the maximum of 275 nm, attributed to π→π* transitions of the aromatic system, along with a shoulder around 350 nm corresponding to n→π* transitions. Upon the addition of metal ions, significant variations in absorption intensity were observed, accompanied by slight spectral shifts (either bathochromic or hypsochromic, depending on the metal ion) [23]. These spectral changes, consistent with modified electronic states, confirm coordination between the metal ions and the nitrogen donor sites of the ligand. The variations in absorption intensity, ranging from enhancement to quenching, suggest varying degrees of complex stability and potential ligand-to-metal charge transfer (LMCT) interactions [47].

Fluorescence emission spectra, recorded at an excitation wavelength of 280 nm, show a distinct emission band centered at 385 nm (Fig. 7b). To quantitatively evaluate the selectivity of Py-NZ, we calculated the fluorescence enhancement factor (F/F_0_) for each metal ion, where F is the emission intensity at 385 nm in the presence of the metal ion and F_0_ is the intensity of the free Py-NZ ligand.

Our analysis reveals that Zn²⁺ induces the most pronounced “turn-on” response, yielding an enhancement factor of 4.1. This value is significantly higher than those observed for Ag⁺ (3.5) and Co²⁺ (2.6), confirming the high selectivity of Py-NZ for Zn²⁺ ions. In contrast, Cu²⁺ acts as a fluorescence quencher (F/F_0_ = 0.9), likely due to its partially filled *d*-orbitals, which facilitate non-radiative energy dissipation and electron transfer processes [48]. The clear differentiation between the Zn²⁺-induced enhancement and Cu²⁺-induced quenching highlights the potential of Py-NZ as a versatile, selective fluorescent probe for applications requiring reliable Zn²⁺ sensing [49].

### Unified spectral analysis of Py–NZ under different HCl concentrations

**Fig. 8:**
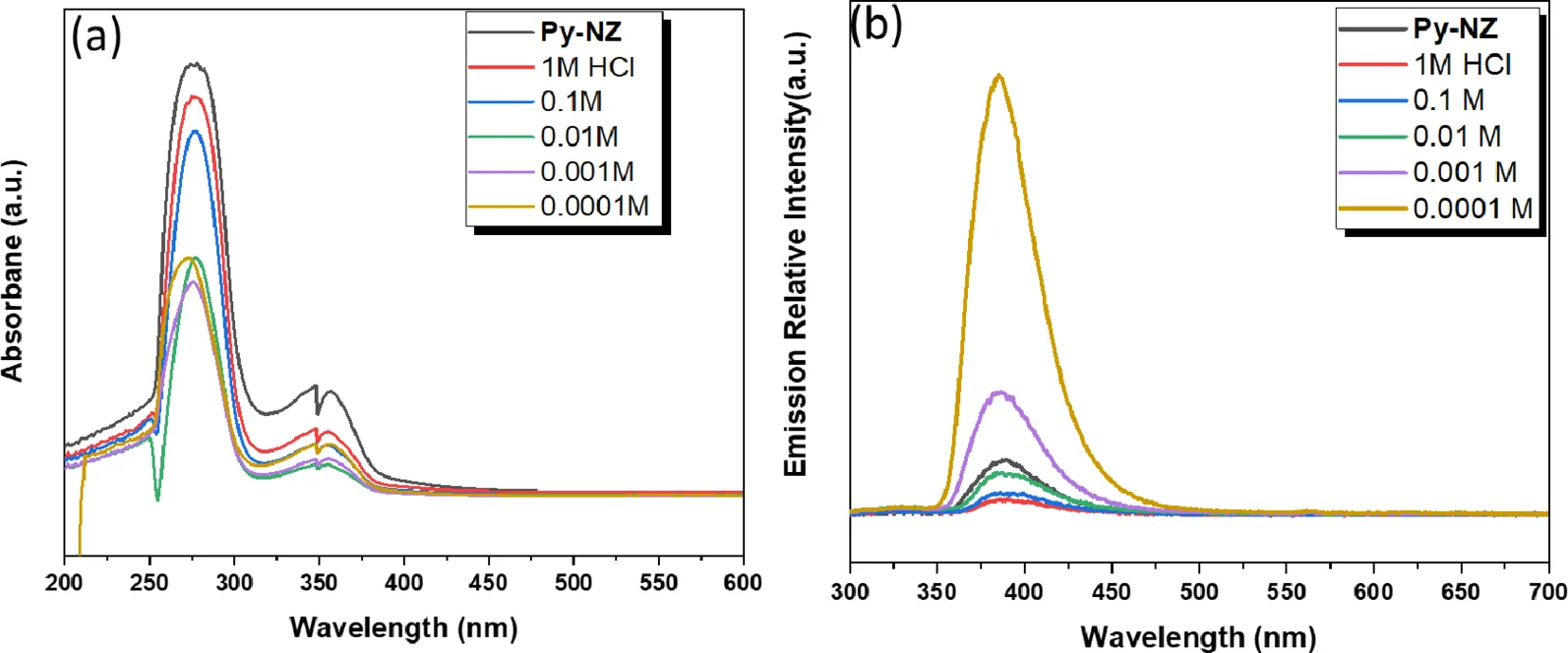
**a** UV–Vis absorption spectra and **b** fluorescence emission spectra (λe_x_ = 280 nm) of Py-NZ in the absence and presence of various concentrations of HCl

To identify acid-induced electronic changes and establish structure–property relationships, the UV–Vis absorption and fluorescence emission spectra of Py–NZ were recorded in the presence of varying HCl concentrations (Fig. 8). The UV–Vis spectra (Fig. 8(a)) exhibit a prominent absorption band centered at approximately 280 nm, attributed to π–π* transitions within the aromatic conjugated framework, together with a weaker band around 350 nm associated with extended conjugation involving the pyridine moiety. Upon gradual addition of HCl, the intensities of both absorption bands decrease progressively, while their positions remain essentially unchanged. This behavior is attributed to protonation of the pyridinyl nitrogen, which converts the pyridine unit into a pyridinium species and reduces the electron-donating contribution of the nitrogen lone pair to the conjugated π-system. Consequently, electron delocalization across the molecule is diminished, leading to lower transition probabilities and reduced absorption intensity. The concentration-dependent attenuation of the absorption bands provides clear evidence of proton-responsive modulation of the electronic structure of Py–NZ [50].

The fluorescence emission spectra show a strong emission band centered at ~ 385 nm (Fig. 8(b)), corresponding to a large Stokes shift of about 105 nm. Such a substantial Stokes shift indicates significant excited-state relaxation and charge redistribution prior to emission, while also minimizing self-absorption effects. Notably, the fluorescence intensity increases progressively as the HCl concentration decreases, indicating that highly acidic conditions induce pronounced fluorescence quenching, whereas enhanced emission is observed at HCl concentrations below 0.001 M. The proton-responsive fluorescence behavior of Py–NZ is consistent with previous reports on pyridine-containing benzoxazine systems. Acid-triggered luminescence modulation has been successfully utilized for the highly sensitive solution-state detection of hydrogen chloride and sulfonic acids (TfOH, TsOH, and MsOH), with detection limits ranging from 1.6 to 9 µM [51]. In such systems, protonation alters the electronic distribution within the molecular framework, thereby affecting the emissive properties.

**Fig. 9:**
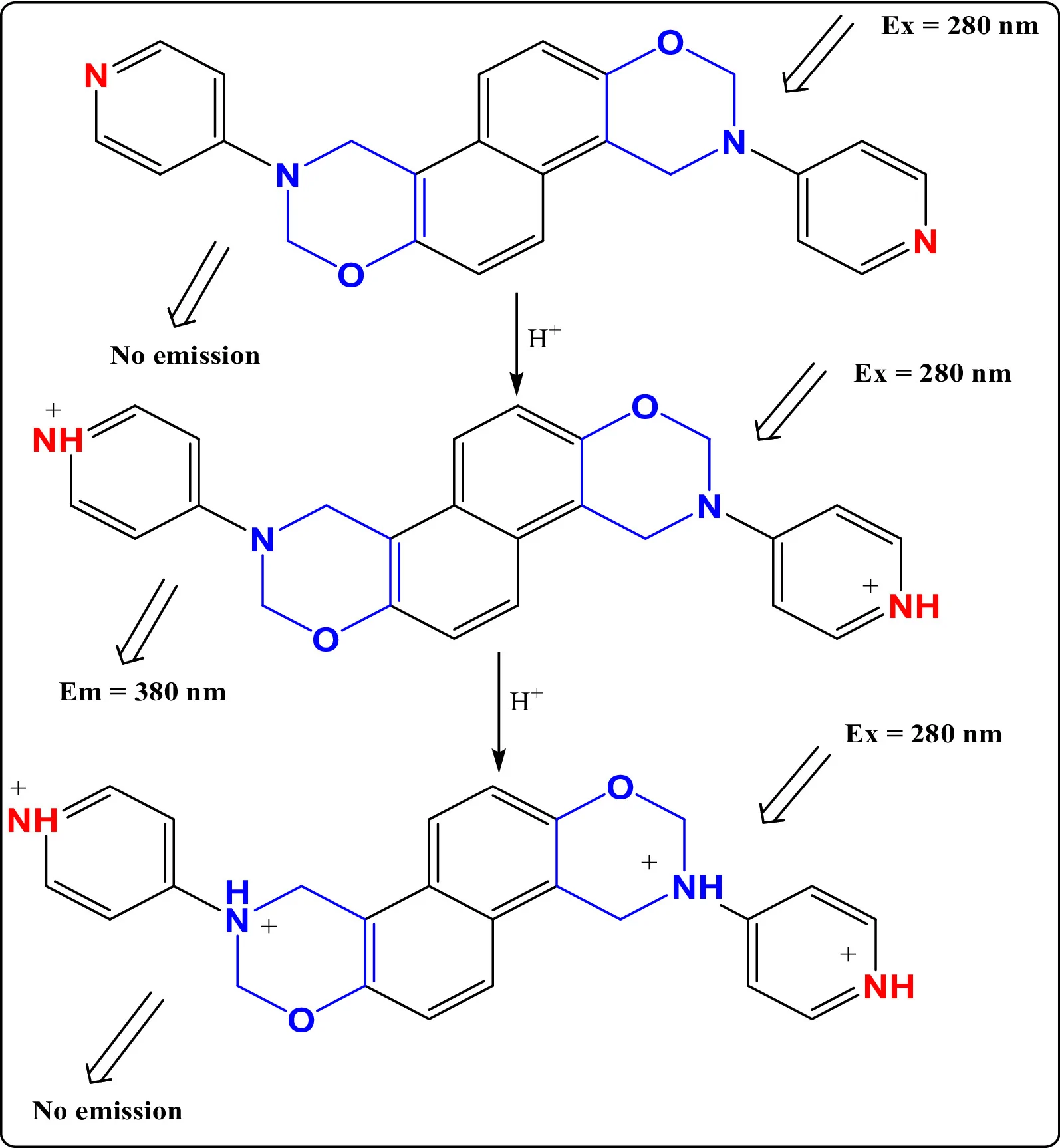
The proposed protonated Py-NZ structure, showing protonation at both the pyridine nitrogen and the benzoxazine ring nitrogen

A probable mechanism for the observed fluorescence quenching under strongly acidic conditions involves the protonation of both the pyridinyl and benzoxazine nitrogen atoms. Previous studies have shown that, at elevated HCl concentrations, protonation of the benzoxazine nitrogen converts the benzoxazine moiety from an electron-donating group into a weak electron-accepting unit. Simultaneously, protonation of the pyridine nitrogen generates a pyridinium species, disrupting the donor–acceptor balance required for efficient fluorescence emission. As a result, the excited-state electronic communication is significantly weakened, leading to substantial suppression of fluorescence (Fig. 9) [52]. Furthermore, extensive protonation facilitates non-radiative deactivation pathways and may promote photoinduced electron transfer processes or internal charge redistribution, which compete effectively with radiative decay and further diminish fluorescence intensity [53]. Upon dilution of HCl, partial deprotonation restores electron density at the nitrogen centers, suppresses these quenching pathways, and enhances the probability of radiative relaxation, resulting in stronger fluorescence emission.

Overall, Py–NZ exhibits a pronounced acid-sensitive fluorescence response, where excessive protonation at high HCl concentrations leads to fluorescence quenching, while lower acidity restores the emissive state and produces significantly enhanced fluorescence intensity. This behavior is important in the development of reversible acid–base fluorescent sensors for detecting and monitoring hydrogen chloride and related acidic environments.

### Energy-dispersive X-ray spectroscopy (EDX) Analysis

**Fig. 10:**
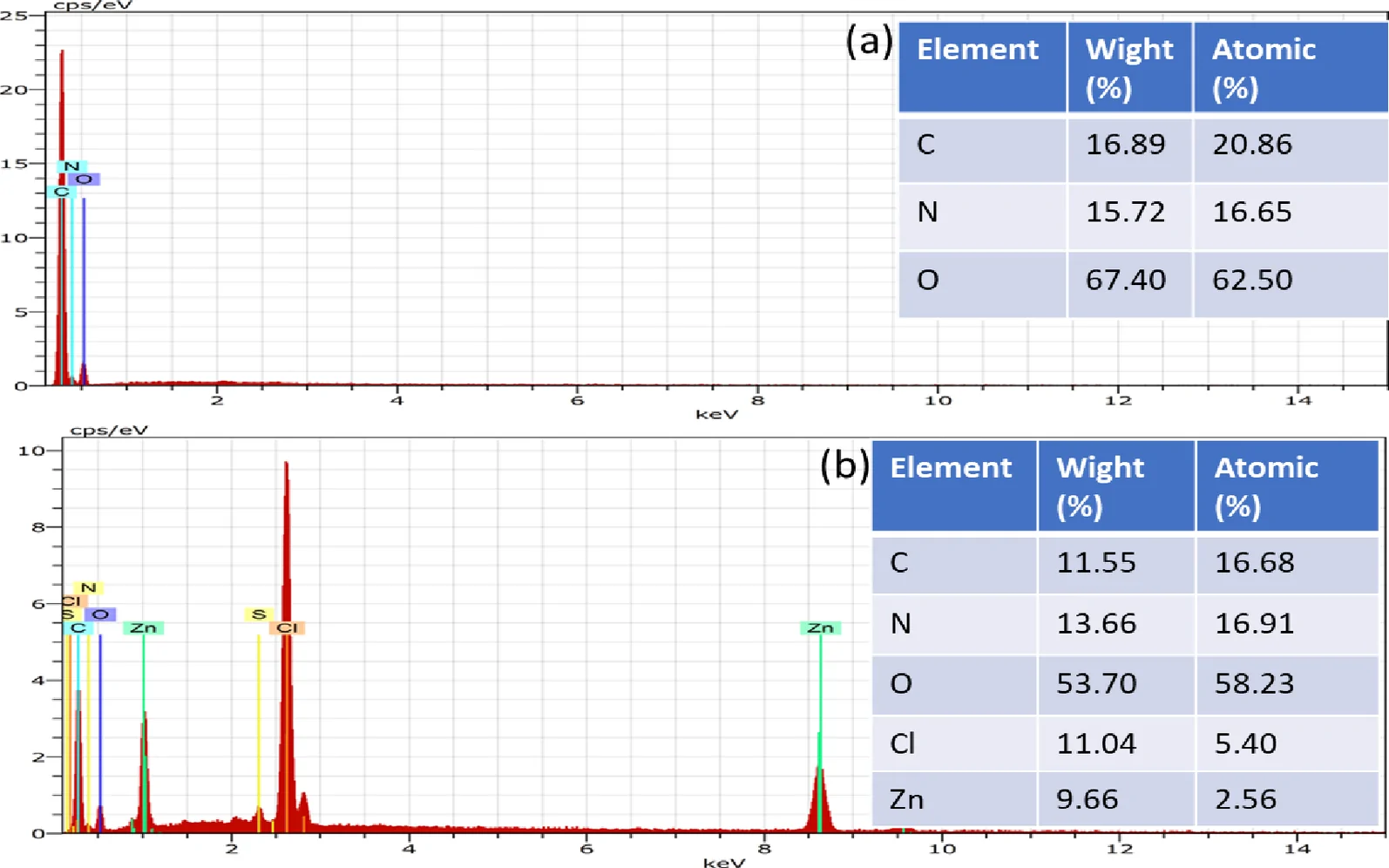
EDX spectra of **a** Poly(Py-NZ), **b** Poly(Py-NZ)/H, **c** Poly(Py-NZ)/Zn

The elemental composition of pure Poly(Py-NZ) and Poly(Py-NZ)/Zn complex samples was investigated by energy-dispersive X-ray spectroscopy (EDX), and the corresponding spectra are shown in Fig. 10. The EDX spectrum of pure Poly(Py-NZ) reveals carbon, nitrogen, and oxygen as the predominant elements, confirming the successful formation of the polymeric framework and the absence of detectable residual impurities. In contrast, the EDX spectrum of Poly(Py-NZ)/Zn exhibits the presence of zinc in addition to carbon, nitrogen, oxygen, and chlorine, confirming the successful complexation of Zn ion with the Poly(Py-NZ) matrix. The detection of chlorine is attributed to coordinated chloride ions and/or residual zinc chloride associated with the metal-complexed structure. Furthermore, variations in the elemental weight percentages, particularly the decrease in the relative carbon and oxygen contents after zinc incorporation, suggest changes in the surface composition resulting from coordination interactions between Zn²⁺ ions and the pyridine nitrogen atoms of the polymer network [54].

The comparatively high oxygen content detected by EDX relative to carbon does not necessarily reflect the actual stoichiometric composition of the polymer. This apparent discrepancy is attributed to the surface-sensitive nature of EDX analysis, where oxygen-containing species, including adsorbed moisture and surface oxidation products, can contribute significantly to the measured signal [55].

### Surface morphology analysis (SEM)

**Fig. 11:**
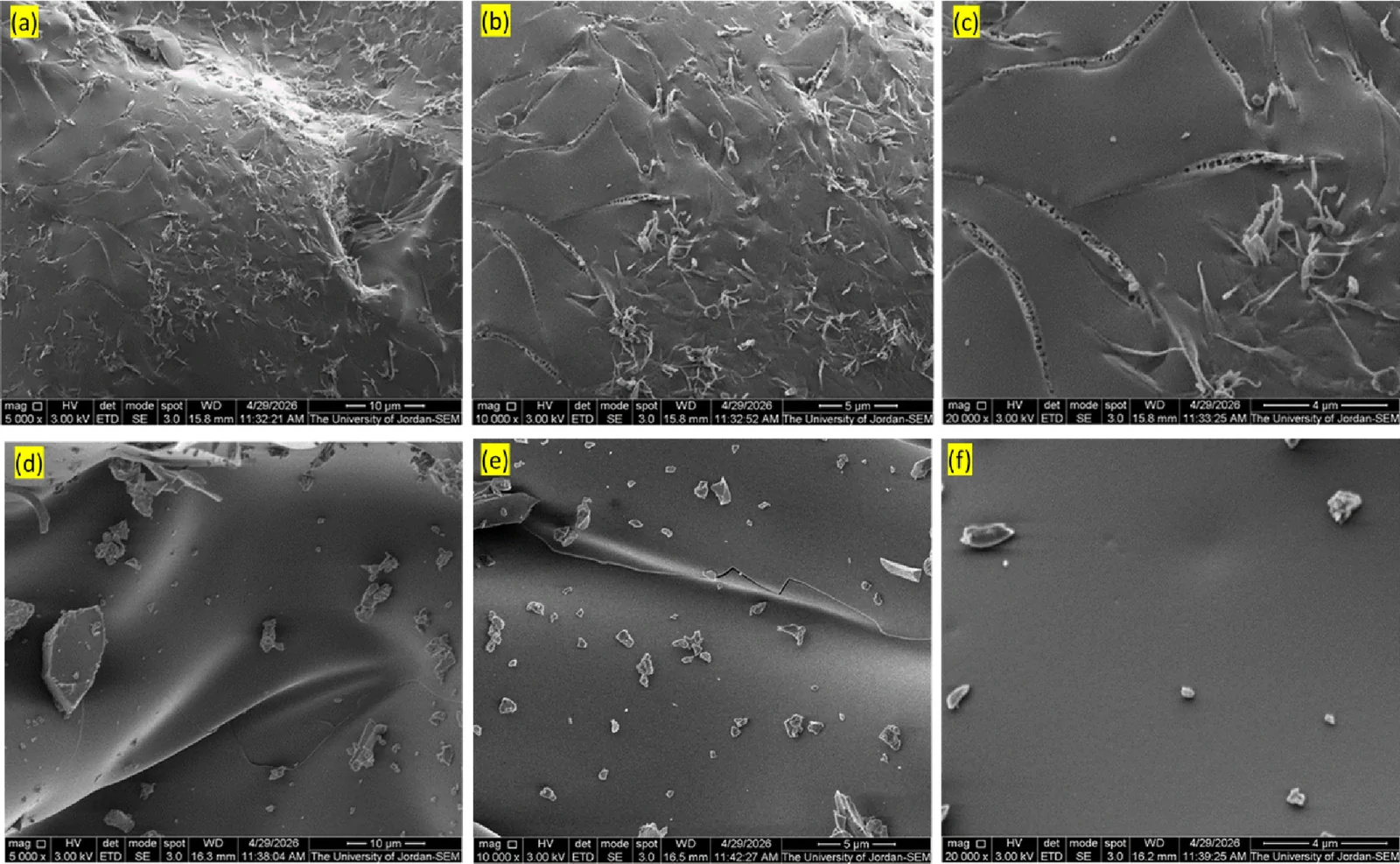
SEM images of **a**–**c** Poly(Py-NZ), and **d**–**f** Poly(Py-NZ)Zn at different magnifications

Scanning electron microscopy (SEM) is a powerful technique for examining the surface morphology of polymeric materials. In this study, SEM was utilized to assess the effect of Zn²⁺ coordination on the microstructure of the pyridine-functionalized PBZ and to elucidate the resulting changes in surface uniformity, compactness, and network architecture. The SEM images of the pyridine-functionalized PBZ, Poly(Py-NZ) (Fig. 11a–c), reveal a rough and heterogeneous surface morphology characterized by irregular folds, wrinkles, and interconnected fibrous-like structures. The surface exhibits noticeable textural variations and localized cavities, suggesting non-uniform chain packing and microstructural defects within the polymer matrix. Such features are commonly observed in crosslinked PBZ networks formed through thermally induced ROP [56].

Following Zn²⁺ coordination, the morphology of Poly(Py-NZ) undergoes a significant transformation (images (d–f)). The coordinated polymer, Poly(Py-NZ)Zn, displays a smoother, denser, and more homogeneous surface with substantially reduced roughness and fewer structural irregularities. The fibrous and wrinkled features observed in the pure polymer are largely replaced by compact and continuous domains, indicating enhanced network organization. This morphological evolution can be attributed to coordination interactions between Zn²⁺ ions and the pyridine nitrogen of the PBZ framework, which act as additional physical crosslinking sites. Consequently, a more compact and ordered structure is formed, leading to reduced defect density and improved surface uniformity [57].

These observations demonstrate that metal–ligand coordination significantly influences the microstructure of the pyridine-functionalized PBZ, resulting in a more densely packed and highly organized polymer network. Furthermore, the improved structural uniformity and stabilization of the polymer framework via metal–ligand coordination can facilitate more reproducible protonation/deprotonation, thereby enhancing sensitivity, stability, and repeatability in acid-responsive sensing applications.

### Antibacterial activity and antioxidant assays

The antibacterial activity results presented in Table 2 demonstrate that all synthesized compounds exhibited moderate antibacterial activity against both tested bacterial strains. Py-NZ and Py-NZ/Zn showed identical MIC values of 31.3 µg/mL against *S. aureus* and 62.5 µg/mL against *E. coli*, indicating that Zn doping did not significantly enhance the antibacterial performance. In contrast, Py-NZ/H exhibited improved activity toward *S. aureus* with a lower MIC value of 15.6 µg/mL, suggesting that protonation with HCl enhanced its antibacterial effectiveness against Gram-positive bacteria. However, all compounds showed weaker activity than the standard antibiotic ciprofloxacin. Additionally, the compounds were generally less effective against E. coli, which may be attributed to the higher resistance associated with the outer membrane structure of Gram-negative bacteria. Uniquely, ascorbic acid antioxidative efficacies could prove reductively scavenging the DPPH radicals into higher stability products with a 0.041 µM IC_50_ value. Collectively tested compounds proved comparably antioxidative to ascorbic acid in the descending order of respective potencies, Py-NZ/Zn > Py-NZ/H > Py-NZ, and within 0.15 < reductive IC_50_ value µM < 70.4 range (Table 2). This indicates that metal complexation (Zn^2+^) and acid protonation (HCl) enhance radical scavenging compared to the parent Py-NZ compound. This improvement may be associated with increased electron delocalization and stabilization of radical intermediates after metal coordination or protonation. In comparison with previously reported benzoxazine-derived antioxidants, the synthesized quinoline-based systems displayed competitive antioxidant activity, highlighting their potential as multifunctional materials combining thermal stability, antibacterial properties, and antioxidant performance [58, 59].

**Table 2:** Minimum inhibitory concentration (MIC) values against *S. aureus* and *E. coli* and DPPH radical scavenging activity of Py-NZ, Py-NZ/Zn, and Py-NZ/H compared with reference pharmacotherapeutic agents

compound	MIC S. aureus (µg/mL)	MIC E. coli (µg/mL)	DPPH- IC_50_ value (µM)
Py-NZ	31.3	62.5	70.40 ± 2.55
Py-NZ/Zn	31.3	62.5	0.15 ± 0.01
Py-NZ/H	15.6	62.5	9.08 ± 0.04
Ciprofloxacin	0.097	0.0008	Ascorbic Acid 0.041 ± 0.0062

Results are mean ± SD (*n* = 4 independent replicates). IC_50_ values (µM) (as Half-maximal inhibitory concentration (IC_50_) refers to the concentration of the inhibitor which is required to inhibit a given biological or biochemical function by half) (concentration at which 50% of DPPH reduction in comparison to non-induced basal incubations calculated within the testing dose range (1.563 or 6.25–200µM) or 50% antiproliferation in comparison to non-induced basal 72 h incubations within 3.125-200 µM range). DPPH: Diphenyl-2-picryl-hydrazyl.

Fundamentally, a Zn²⁺ complex with naphthoxazine functionalized with pyridine serves as a strong bio-inorganic hybrid. The Zn²⁺ functions as a redox-inactive Lewis acid, maintaining system stability without producing damaging free radicals, whereas the naphthoxazine-pyridine ligand offers robust radical-scavenging and UV-absorbing properties [60].

Prominently, Naphthoxazine cores often display hydroxyl or methoxy substituents. Their conjugated frameworks enable them to readily transfer electrons or hydrogen atoms to counteract Reactive Oxygen Species (ROS). Profoundly Zinc (II) ions contribute to enhancing the body’s natural antioxidant defense mechanisms. Substantially aligning the metal with a pyridine-modified naphthoxazine can produce a very stable metal-organic framework that enables specific antioxidant or photoprotective effects. These complexes are vastly studied in the fields of pharmaceuticals and high-end cosmetics (such as sophisticated sunscreens) due to their combined photoprotective and antioxidant properties [61].

## Conclusion

In this study, a novel pyridine-functionalized naphthoxazine (Py-NZ) was successfully synthesized via a one-pot Mannich condensation and fully characterized by NMR, FTIR, and HRMS. The cured Poly(Py-NZ) exhibited exceptional thermal stability, with a char yield of 69% at 800 °C and a limiting oxygen index (LOI) of 45.1, indicating it as a high-performance flame-retardant material. Systematic investigation of Py-NZ’s optical responses revealed that metal coordination and protonation significantly modulate its photophysical properties. Specifically, Zn²⁺ coordination induced pronounced fluorescence enhancement due to its d¹⁰ configuration, whereas Cu²⁺ led to a little quenching via non-radiative decay, compared with a pristine Py-NZ. Additionally, Py-NZ demonstrated acid-sensitive fluorescence: high HCl concentrations quenched the emission through protonation-enhanced non-radiative pathways, while lower acidity restored radiative emission. These findings establish Py-NZ as a dual-responsive fluorescent chemosensor for both metal ions and protons. Biologically, Py-NZ and its modifications showed moderate antibacterial activity against Staphylococcus aureus and Escherichia coli, with Py-NZ/H exhibiting enhanced activity against Gram-positive bacteria (MIC = 15.6 µg/mL). Furthermore, in the DPPH assay, all tested compounds displayed antioxidant activity comparable to ascorbic acid, with Py-NZ/Zn complex showing the highest potency (IC₅₀ = 0.15 ± 0.01). The Poly(Py-NZ) core contains O–H group capable of donating a hydrogen atom to the DPPH radical, converting it to reduced DPPH–H. Zn coordination withdraws electron density from the phenolic oxygen, thereby increasing O–H bond polarization and reducing its bond dissociation energy, which facilitates more efficient hydrogen atom transfer. The resulting oxygen-centered radical is further stabilized through extensive resonance delocalization over the conjugated naphthalene π-system.

## Supplementary Information

Below is the link to the electronic supplementary material.

Supplementary Material 1.

## Data Availability

All data generated or analyzed during this study are included in this article.
