# Supplementary material for: A novel pyridine-functionalized naphthoxazine: thermal stability, proton and metal ion-responsive optical sensing, and biological activity with DFT validation
Source: BMC Chem. 2026 Jul 27;20(1):208. doi: 10.1186/s13065-026-01887-x (PMC13689125; doi:10.1186/s13065-026-01887-x)
Supplement: Supplementary file 1 [file 13065_2026_1887_MOESM1_ESM.docx]

**A Novel Pyridine-Functionalized Naphthoxazine: Thermal Stability, Proton and Metal Ion-Responsive Optical Sensing, and Biological Activity with DFT Validation**

Abdulsalam Mahdy ^a,b,c^*, Jalal A. Zahra ^c^, Randa N. Haddadin ^d^, Violet Kasabri ^e^, Murad A. AlDamen ^c *^

^a^Chemistry Department, Faculty of Education & Science, Rada'a, Albaydha University, Albaydha, Yemen.

^b^Department of Pharmacy, Faculty of Medicine and Health Sciences, Ar-Rasheed Smart University, Sana’a, Yemen.

^c^Chemistry Department, School of Science, The University of Jordan, Amman, 11942, Jordan.

^d^ Department of Pharmaceutics & Pharmaceutical Technology, School of Pharmacy, The University of Jordan, Amman, 11942, Jordan.

^e^ Department of Biopharmaceutics and Clinical Pharmacy, School of Pharmacy, The University of Jordan, Amman, 11942, Jordan.

* Corresponding author:

E-mail addresses: [abdualsalam735@gmail.com](mailto:abdualsalam735@gmail.com) (A. Mahdy) and [maldamen@ju.edu.jo](mailto:maldamen@ju.edu.jo)

(Murad A. AlDamen),

**Stock Solutions**

Stock solutions of Py‑NZ (1 × 10⁻^5^ M) and metal salts (1 × 10⁻^4^ M) were prepared in DMSO. Metal stock solutions were prepared at higher concentrations so that a single small aliquot could be added to the Py‑NZ solution, keeping the DMSO content in the cuvette below 2% (v/v).

**Preparation of Py-NZ/M Complexes**

UV–Vis and photoluminescence (PL) measurements were performed using 2 mL of **Py-NZ** solution (1 × 10⁻⁵ M) in a clean cuvette. A **single aliquot of metal stock solution** was added to achieve the desired Py-NZ -to-M ratio. The volume of the aliquot was calculated to reach the target metal concentration in the cuvette. The solution was slightly mixed using a micropipette to avoid air bubbles. All measurements were performed immediately after metal addition to monitor complex formation accurately.

**UV–Vis Spectroscopic Measurements**

The absorption spectra of pure Py-NZ and the Py-NZ/M complexes were recorded in the 200–800 nm range to evaluate changes in electronic absorption upon complexation with various metal ions. Additionally, UV spectra of Py-NZ/HCl were recorded at different HCl concentrations.

**Photoluminescence (PL) Measurements**

PL spectra were recorded using an excitation wavelength of 280 nm. The emission spectra of the Py-NZ/M complexes were compared with those of free Py-NZ to assess fluorescence quenching or enhancement in the presence of various metal ions. Additionally, PL spectra of Py-NZ/HCl were measured at different HCl concentrations. The DMSO content was maintained below 2% (v/v) to minimize solvent effects on the PL response

Fig. S1. Photographs of Py-NZ monomer and its pyridinium salt

Fig. S2. Mass spectrum of Py-NZ monomer

Figure S3. COSY NMR spectrum of Py-NZ monomer

Figure S4. HMQS NMR spectrum of Py-NZ monomer

Fig. S5. HMBC NMR spectrum of Py-NZ monomer

Fig. S6. ^1^H-NMR spectra of Py-NZ/Zn Poly(Py-NZ)/H

**Figure S7.** FTIR spectrum of Py-NZ/Zn
